# 

**Published:** 1895-08-10

**Authors:** 


**Notices of Births, Deaths, and Marriages** are inserted in this column at a charge of 2s. 6d. for an announcement not exceeding four lines.

**Notice to Advertisers and Subscribers.**

All Advertisements, Orders for Copies of The Hospital and Business Communications should be addressed to **The Manager, NOT TO THE EDITOR,** The Hospital, 428, Strand, London, W.C.

The Cost of Subscription, post free, is as follows:—Per week, 2½d.; per quarter, 2s. 8d.; per half-year, 5s. 3d.; per annum, 10s. 6d. Foreign:—Per Annum, 15s. 2d.; payable, in all cases, in advance.

### NOTICE TO CORRESPONDENTS.

All MS., Letters, Books for Review, and other matters intended for the Editor should be addressed The Editor, The Lodge, Porchester Square, London, W.

The Editor cannot undertake to return rejected MS., even when accompanied by stamped directed envelope.

**"Burdett's Hospital Annual," 1895.**

Sixth year of publication. 950 pp. Scarlet cloth, gilt lettered. Price 5s. A most complete guide to British, Colonial, Indian, and American Hospitals, Dispensaries, Nursing and Convalescent Institutions, and Asylums. A few copies of the "Annual" for 1894 may still be obtained on application to the Publishers, The Scientific Press (Limited), 428, Strand, London, W.C.

**NOTICE.—Vol. XVII. of "The Hospital" (October, 1894, to April, 1895), in handsome cloth binding, is on sale at the Office of this Journal. Price 6s. Cases for binding the Numbers contained in this Volume, 1s. 6d. Index to Vol. XVII., 2½d., post free.**

**The Monthly Part for August is now ready, price 6d.; by post, 8d.**

### Scraps and Gleanings.

The new wing to Gravesend Hospital has been formally opened.

The Invalid Children's Aid Association will henceforth co-operate in their work with the Charity Organisation Society.

The Grocers' Company have voted £100 and the Mercers' Company £52 10s. in aid of Queen Charlotte's Lying-in Hospital, Marylebone.

The Deighton, Sheepridge, and surrounding districts have just held their fourteenth annual demonstration in aid of the Huddersfield Infirmary.

The number of patients treated during the year at the Belfast Hospital for Diseases of the Skin has been 938, bringing up the total since the foundation of the hospital to 29,347.

The anti-rabic records of the Pasteur Institute show that 1,387 persons were treated during the year, seven of whom died. The number of English patients rose from 25 in 1893 to 128 in 1894.

The Hospital Saturday Parade, under the auspices of the Brighton friendly and trade societies, took place last week; as also the eleventh annual demon tration of the Stockton and Thornaby friendly and trades societies, both respectively on behalf of the local hospitals and charities.

The Rotherham Hospital and Dispensary Committee, upon losing the services of Dr. Reid, passed a resolution acknowledging his services by a donation from the funds. Dr. Reid, during the three years' tenure of his office, has initiated a course of lectures for probationers at the hospital.

The position of financial affairs at the South Devon and East Cornwall Hospital has become so serious that the committee have been forced to pass a resolution reducing the number of patients daily under treatment to a maximum of 100. This limitation will only continue in force until increased funds are placed at the disposal of the committee

The twenty-first annual meeting of the Liverpool Convalescent Institution was held last week in the establishment at Woolton. The report states that the number of free beds has been increased from eight to ten. The debit balance has been reduced from £435 to £156, but this has been achieved by utilising for present needs, amounts which might otherwise have been left out at investment.

### THE STUDENT OF MEDICINE.

### VACANCIES.

The following vacancies are announced this week, the amount of salary in each case being given after the name of the institution:—

### Resident Medical Officers.

Cumberland Infirmary, Carlisle.—House Surgeon. Salary £70 per annum, with board, lodging, and washing. Appointment for one year. Applications to the Secretary by August 24th.

Chichester Infirmary.—House Surgeon. Salary £80 per annum, with board, lodging, and washing. Applications to the Secretary by August 12th.

Durham County Asylum.—Pathologist and Junior Medical Officer. Salary £100 per annum, with board and lodging. Applications to the Superintendent, Durham County Asylum, Winterton, Ferryhill, by August 10th.

Devon and Exeter Hospital, Exeter.—Assistant House Surgeon; doubly qualified and unmarried. Salary £40 per annum, with board and lodging, not including alcoholic liquors and aerated waters. Applications to Albert E. Boyce, Secretary, by August 10th.

East Suffolk and Ipswich Hospital, Thoro'fare, Ipswich.—Second House Surgeon; unmarried; doubly qualified. Salary £70 per annum, with board, lodging, and washing. Applications to the Secretary by August 13th.

Hulme Dispensary, Dale Street, Stretford Road, Manchester.—House Surgeon; doubly qualified. Salary £130 per annum, with apartments, attendance, coal, and gas. Applications to the Honorary Secretary Medical Committee by August 14th.

Liverpool Infirmary for Children.—Assistant House Surgeon. Appointment for six months. Board and lodging provided, but no salary. Applications to C. W. Carver, Honorary Secretary, by August 10th.

Lincoln County Hospital.—Assistant House Surgeon. Appointment for six months, but eligible for re-election. An honorarium of £10 for each period of six months, subject to the approval of the Weekly Board, with board, residence, and washing. Applications to the Secretary by August 10th.

### Non-Resident Medical Officer.

Royal Ear Hospital, Frith Street, Soho Square, W.—Assistant Surgeon. Applications to Donald Murray, Honorary Secretary, by August 10th.

### UNIVERSITIES AND COLLEGES.

### University College, Dundee.

The trustees of the late Miss Margaret Harrls have determined to carry out the recommendation of the Universities' Commissioners to establish a chair of Physics at Dundee. A number of first-class securities, valued at £14,000, and yielding £470 a year, are to be set apart to provide the salary of the professor and some of the expenses of the chair. The new professor will be appointed before the beginning of next session, and the salary will be £400, with a share of the fees.

The trustees are to reserve the balance of Miss Harris's estate, amount-to some £16,000, and to apply the interest of it from time to time for College purposes.

### The Conjoint Board in Scotland.

The quarterly examinations in Edinburgh for the Triple Qualification of the Royal College of Physicians, Edinburgh, Royal College of Surgeons, Edinburgh, and Faculty of Physicians and Surgeons, Glasgow, took place in July, with the following results:—

First Examination, Four Years' Course.—Of 14 candidates, the following seven passed: T. Donovan, E. B. Anderton, D. Nyhan, J. W. Dickson, M. P. Rodgers, J. J. Anthony-Pillay, C. H. Bennett. Three candidates entered for divisions and passed in one division each.

Five Years' Course.—Of 28 candidates, the following 15 passed: G. F. Stooke (with distinction), W. F. Oliver, J. Bygott, C. Jones, G. W. Hardie, E. A. Boxer, J. S. Hamilton, T. Neville, H. J. Clarke, J. H. Gibbs, Hilda Maud McFarlane, Jeannie Hamilton Traill, Emma Bond, G. A. Paulin, J. McGrath. Twenty-four candidates entered for divisions and 16 passed in one or two divisions.

Second Examination, Four Years' Course.—Of 17 candidates, the following 12 passed: G. J. Goldie, B. J. Nolan, P. Brady, J. J. Fuller, P. B. Molony, W. F. Campbell, S. S. Broadbent, J. A. Holmes, R. S. Wells, B. S. Sanders, J. A. Finch, and W. G. Cook. Ten candidates entered for divisions and four passed.

Five Years' Course.—Of 19 candidates the following 14 passed: R. Mackie, Rosalie Berthon, Elsie Rosa Cresswell Taylor, Matilda Hetty Grace Russell, J. McGrath, C. F. Ackland, E. H. Sheldon, J. Cotter, T. J. O'Donovan, W. S. Soutar, E. L. Munn, Margaret Grant Campbell Brodie, J. Owens, and J. K. Calder.

**THE STUDENT OF MEDICINE**—*continued.*

Third Examination, Five Years' Course.—Of seven candidates the following four passed: E. B. Kitching, A. Cameron, W. Robertson, and J. Dodds.

Final Examination.—Of ninety-five candidates examined for the whole examination the following thirty-nine passed, and were admitted L.R.C.P.E., L R.C.S.E., and L.F.P. and S.G.: E. A. Purcell, M. T. Archdall, F. G. Bennett, A. E. Clayton, Laura Elizabeth Forster, Margaret Penelope Munro, P. K. Chitale, C. B. Rossiter, A. L. Levy, J. A. B. Colaco-Belmonte, G. Singh. P. Sullivan, E. F. de Jong, J. W. N. Hudson, K. P Popat, Sarah B. McMordie, T. J. Kennedy, Lydia Ann Leney, J. Craig, H. Paine; E. H. Guvand, Mary Harriet Simson, W. Craig, A. K. Ludlam, Agnes Lillie Cousins, H. W. Lloyd, H. H. Gourley, C. C. Bullmore, H. Winstanley, W. A. McCutchan, T. Murphy, J. H. Waddington, J. C. Forbes, A. T. Savage G. A. I. Mackay, E. A. R. Laing, A. Speight, Helen Lauder, and E. P. Hasluck. Twenty-two candidates entered for divisions; and the following completed the examination and received the diplomas: R. M. Johnson, W. Morris. One candidate passed in one division.

### University of Glasgow.

List of Degrees Conferred on July 25th, 1895.

DOCTORS OF MEDICINE (M.D.).

I. With Honours.—T. K. Monro, M.A., M.B., C.M.

II. With Commendation.—C. Banks, M.B., C.M.; A. L. Bell, M.B., C.M.; F. S. Campbell, M.B., C.M.; J. Charles, M.B, C.M.; J. M'Gregor, M.B., C.M.

III. Ordinary Degree.—P. Gardiner, M.B., C.M.; A. G. Hay, M.A., M.B., C.M.; T. T. Macklin, M.B.; J. M. McPhail, M.A., M.B., C.M.; J. J. Robb, M.B., C.M.; J. Smith, M.B., C.M.; J. A. Wilson, M.B., C.M.

BACHELORS OF MEDICINE AND MASTERS IN SURGERY (M.B., C.M.).

I. Honours.—*J. Ferguson, M.A., H. A. Pattullo, E. A. Walker, M.A.

II. High Commendation.—W. Scobie, B.D., A. H. Stewart, R. Howie, H. A. Watson, M.A., W. H. Lang, B.Sc., A. Young, B.Sc.

III. Commendation.—R. B. Barr, A. Stevenson, S. D. Cowan, M.A., W. Lawson, F. Macrae, R. M. Fraser, M.A., Emmeline Marie Stuart, R. K. Miller, M. Watson, J. L. Anderson, A. C. Muir, T. Bell, F. B. Cormick, G. Mowat, W. D. Miller, W. Alexander, J. M'Cash.

IV. Ordinary Degrees.—J. C. Abbott, J. Adam, W. Allan, A. R. Anderson, J. Anderson, J. J. S. Anderson, T. Armstrong, W. S. Baird, F. J. Barker, M.A., A. Blair, H. A. Böedeker, W. Burns, T. Campbell, V. E. Chang, W. Clow, M.A., B.Sc., J. Cumming, H. Davies, J. Divine, W. Donaldson, M. Dunning, R. J. Edwards, S. English, J. Findlay, W. D. Findlay, A. A. Finkelstein, J. L. Forrest, J. Forster, J. R. Foulds, A. F. Galloway, T. C. Garrett, J. Gillan, M.A., W. Graham, W. Grove, W. Hay, B.D., A. Holt, A. B. Hughes, J. Jack, J. W. Jackson, G. Jubb, D. Kerr, W. J. Kerr, D. C. Kirkhope, J. Kirkwood, D. Lewis, J. D. Loutitt, J. A. D. Mulholland, D. Munro, D. M'Cormick, D. M'Donald, W. J. Mackinnon, L. MacLachlan, H. M'Laren, M. N. MacLay, W. Macleod, G. M'Pherson, M.A., A. R. Oliver, E. J. Primrose, M.A., J. B. Rae, J. Rankin, J. N. Robertson, J. Sandilands, J. Smith, J. Sproull, H. Stevenson, A. Stewart, J. Stewart, J. Stirling, H. W. Thomson, C. K. Toland, F. Wolverson.

* Mr. Ferguson gains the Brunton Memorial Prize of £10, awarded to the most distinguished Graduate of Medicine of the year.

### Medical Appointment.

EAST SUFFOLK AND IPSWICH HOSPITAL.

